# Epigenetic Deregulation of Apoptosis in Cancers

**DOI:** 10.3390/cancers13133210

**Published:** 2021-06-27

**Authors:** Ezgi Ozyerli-Goknar, Tugba Bagci-Onder

**Affiliations:** 1 Brain Cancer Research and Therapy Laboratory, Koç University School of Medicine, Istanbul 34450, Turkey; eozyerli14@ku.edu.tr; 2 Research Center for Translational Medicine, Koç University, Istanbul 34450, Turkey

**Keywords:** apoptosis, evasion, cancer, chromatin modifying enzymes, epigenetic drugs, therapy

## Abstract

**Simple Summary:**

Disruption of the balance between cell division and cell death (apoptosis) is an extremely important factor contributing to cancer formation and progression. While cancer cells acquire characteristics to divide uncontrollably, they also adopt several mechanisms to escape apoptosis. These mechanisms appear through genetic alterations, by selection of new mutations. However, perhaps a more favorable approach for cancer cells is suppressing apoptosis at the gene expression level, through epigenetic alterations. In this review, we present an overview of how the apoptotic program components within cancer cells are deregulated at an epigenetic level. We also provide an update on the potential use of epigenetic modifier drugs (Epi-drugs) as anti-cancer agents.

**Abstract:**

Cancer cells possess the ability to evade apoptosis. Genetic alterations through mutations in key genes of the apoptotic signaling pathway represent a major adaptive mechanism of apoptosis evasion. In parallel, epigenetic changes via aberrant modifications of DNA and histones to regulate the expression of pro- and antiapoptotic signal mediators represent a major complementary mechanism in apoptosis regulation and therapy response. Most epigenetic changes are governed by the activity of chromatin modifying enzymes that add, remove, or recognize different marks on histones and DNA. Here, we discuss how apoptosis signaling components are deregulated at epigenetic levels, particularly focusing on the roles of chromatin-modifying enzymes in this process. We also review the advances in cancer therapies with epigenetic drugs such as DNMT, HMT, HDAC, and BET inhibitors, as well as their effects on apoptosis modulation in cancer cells. Rewiring the epigenome by drug interventions can provide therapeutic advantage for various cancers by reverting therapy resistance and leading cancer cells to undergo apoptotic cell death.

## 1. Introduction

**Figure 1:**
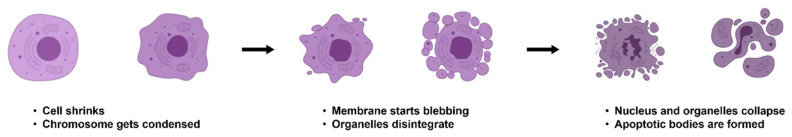
**Morphological changes during apoptosis.** Cells undergoing apoptosis start to shrink, which is followed by chromosome condensation and disintegration of organelles. Cells later collapse into apoptotic bodies that are further eliminated by the immune system. Figure generated at Biorender.com accessed on 20 April 2021.

Apoptosis is a form of programmed cell death (PCD) that is observed in multicellular organisms, and it serves as a barrier to suppress cancer development [1]. Apoptosis is essential to maintain tissue homeostasis through elimination of damaged, infected, or aged cells. The decision to undergo apoptosis is made on the basis of the cells’ interpretation of environmental stimuli or their self-assessment of cellular damage. When induced to undergo apoptosis, cells exhibit specific structural changes characterized by membrane blebbing, nuclear fragmentation, cell shrinkage, chromatin condensation, chromosomal DNA fragmentation, and formation of apoptotic bodies [2] (Figure 1).

This physiological program is tightly controlled within the cells, with many molecular players involved. It is well established that, in cancer, cells adopt mechanisms that allow them to evade apoptosis [3]. While genetic alterations are key in apoptotic evasion [4], epigenetic adaptation through silencing or activating molecular players of apoptosis also contributes to this process [5]. Most epigenetic changes are governed by the activity of chromatin-modifying enzymes that add, remove, or recognize different marks on histones and DNA [6]. In this review, we elaborate on how apoptosis signaling components are deregulated at epigenetic levels, particularly focusing on aberrant DNA methylation, aberrant histone methylation, and regulation by miRNAs. We also discuss the advances in cancer therapies with “Epi-drugs” that modulate the activity of chromatin-modifying enzymes and alter the expression and/or the activity of apoptotic molecules in cancer cells.

## 2. Mechanisms of Apoptosis

Apoptosis can be triggered in response to several external stimuli such as chemicals or radiation, leading to excessive intracellular stress and DNA damage [7]. It is a very tightly regulated process governed by the activity of dozens of proteins, the most significant of which are caspases (cysteine-aspartic proteases), responsible for proteolytic degradation of cellular components. Apoptotic caspases are subcategorized as initiators (caspase-2, -8, -9, -10) and executioners (caspase-3, -6, -7). Once activated by the initial apoptotic signal, initiator caspases cleave and activate executioner caspases, which degrade cellular components and cause apoptosis-related changes in cellular morphology [8].

Depending on the source of the apoptotic stimuli, either the intrinsic or the extrinsic pathway of apoptosis gets activated. The intrinsic pathway is triggered by intracellular signals in response to cellular genotoxic stress inducers, such as DNA damage, defective cell cycle, loss of extracellular matrix attachment, hypoxia, or deprivation of cell survival factors. These stimuli lead to mitochondrial outer membrane permeabilization (MOMP) that initiates mitochondrial release of proapoptotic factors, such as cytochrome C, apoptosis-inducing factor (AIF), and Smac/DIABLO from the mitochondrial intermembrane space [9,10]. Released cytochrome C then combines with apoptotic protease-activating factor 1 (Apaf-1) to form the large apoptosome complex, which triggers autoactivation of caspase-9 and consequent stimulation of effector caspases, caspase -3, -6, and/or -7 [11]. Smac binds and blocks the inhibitor of apoptosis proteins, IAPs (IAP-1, IAP-2, XIAP, NIAP, BRUCE, and Survivin), further promoting caspase-9 activation. Mitochondrial membrane permeability and consequent release of these factors are strictly regulated by pro- and antiapoptotic Bcl-2 family proteins [12,13]. Proapoptotic Bcl-2 family members are subdivided into two categories according to the number of BH (Bcl-2 homology) domains they contain (named BH1, BH2, BH3, and BH4 domains). Bax and Bak proteins possess several BH domains, whereas proteins such as Bid, Bad, Bim, Bmf, Puma, and Noxa have only the BH3 domain. These BH3-only proteins are responsible for activation of Bax and/or Bak through initiating their oligomerization and insertion into outer mitochondrial membrane, thereby allowing the permeabilization process [14]. On the other hand, antiapoptotic Bcl-2 family members, such as Bcl-2, Bcl-XL, Mcl-1, A1, and Bcl-W inhibit the Bax/Bak-mediated pore formation on mitochondria through binding and retro-translocating Bax/Bak from the mitochondria back into the cytosol [15]. That retro-translocation process is blocked by BH3-only protein binding and inactivation of antiapoptotic Bcl-2 family members. Therefore, the delicate balance of the expression and activity levels of pro- and antiapoptotic Bcl-2 family proteins is extremely important for the control of apoptosis.

**Figure 2:**
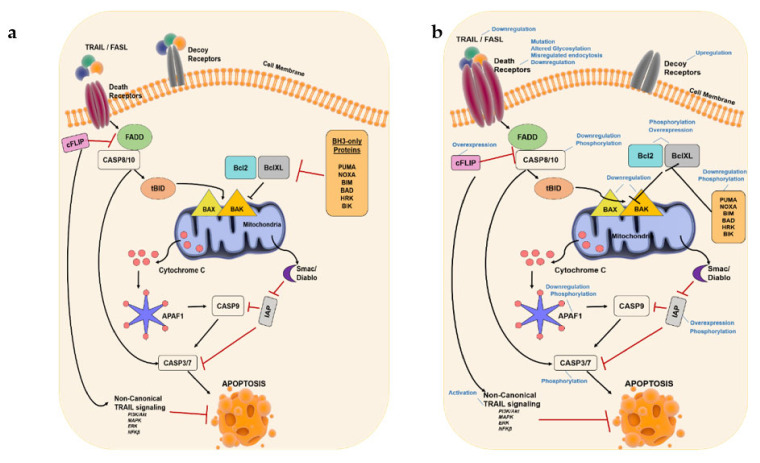
**Extrinsic and intrinsic apoptosis and its deregulation in cancer cells.** (**a**) Extrinsic apoptosis inducers, such as TRAIL or FasL, bind to death receptors and lead to FADD-mediated caspase-8 activation. Active caspase-8 cleaves and activates effector caspase-3/7 and leads to apoptosis. Caspase-8 also truncates Bid and causes Bax and Bak oligomerization in mitochondrial outer membrane that leads to cytochrome C release, consequent activation of caspase-9 and effector caspases-3/7, and apoptosis (intrinsic). Intrinsic apoptosis can also be triggered by BH3-only proteins, which inhibit Bcl-2 and Bcl-xL antiapoptotic proteins, facilitating Bax and Bak activity. XIAP and cFLIP inhibit apoptosis through interfering with caspase activation. (**b**) Mechanisms of apoptosis evasion in cancer cells. The balance of pro- and antiapoptotic signal mediators is deregulated both transcriptionally (e.g., DNA hyper/hypomethylation) and post-translationally (e.g., phosphorylation) in cancer cells. Figure generated at Biorender.com accessed on 20 April 2021.

Proapoptotic Bcl-2 family protein expressions are tightly controlled by p53 tumor suppressor, which partially explains the potency of DNA-damaging agents to induce intrinsic apoptosis [16]. On the contrary, the extrinsic pathway gets activated by extracellular signals transmitted to the cell with the help of proapoptotic ligands of the TNF (tumor necrosis factor) family, such as CD95L/FasL or TRAIL/Apo2L [17], binding to their cell surface death receptors, CD95/Fas and DR4/DR5, respectively [18]. Death domains at the carboxyl terminus of ligand-bound active receptors recruit Fas-associated protein with death domain (FADD), which further recruit initiator caspases (caspase-8 or -10) through the death effector domain (DED). Consequently, death-inducing signaling complex (DISC) is formed [19], and initiator caspases are activated by autocleavage. These further activate effector caspases, caspase-3, -6, and/or -7, and facilitate apoptosis through degradation of cellular components [20]. Extrinsic apoptosis can be inhibited by decoy receptors, which lack the catalytic domain necessary for proper apoptosis induction [21], as well as by c-FLIP [22]. The c-FLIP protein has a homologous sequence with caspase-8 and, thus, can compete for binding to FADD and consequently form a distinct signaling complex that activates NF-κB, PI3K, and MAPK signaling pathways [23]. These pathways play important roles in cell survival and proliferation and counteract the apoptotic activity. While the transduction of extrinsic or intrinsic apoptotic signals involves the activation of several proteins in a linear mode, a major crosstalk between extrinsic and intrinsic apoptosis is mediated by the protein Bid. Upon its cleavage by initiator caspase-8 [24], truncated Bid, now called “tBid”, oligomerizes Bak or Bax into mitochondrial pores, changing mitochondrial membrane polarization and causing the release of cytochrome C and Smac [25,26]. Therefore, Bid links the two apoptotic pathways (Figure 2a).

Cells are categorized as type I or II according to the type of apoptotic machinery they utilize. Type I cells rely solely on extrinsic apoptosis pathway without the involvement of mitochondrial signaling, since active caspase-8 produced by the DISC complex is adequate to directly activate the effector caspases and promote apoptosis [27]. On the other hand, in type II cells, both extrinsic and intrinsic pathways are utilized with the help of Bid, which amplifies effector caspase activation [28]. Most cells are known to be type II, whereas some cell types, such as mesenchymal cells, can be type I.

Although apoptosis is the most well-characterized and evolutionary conserved PCD up to date, there are several alternative mechanisms of regulated cell death, such as necroptosis, pyroptosis, and ferroptosis [29]. Pyroptosis is driven by the activation of the inflammasome, ferroptosis is iron- and lipotoxicity-dependent, and necroptosis involves kinase-mediated signaling (e.g., RIPK, MLKL) induced by extracellular (death receptor activation of death receptors or Toll-like receptors) or intracellular signals (microbial nucleic acids) [30]. Apoptosis serves as an anti-inflammatory response preserving homeostatic integrity, since intact apoptotic bodies are eliminated by resident phagocytic cells [31]. On the other hand, necroptosis and pyroptosis trigger inflammation due to the loss of plasma membrane integrity and release of intracellular contents [32,33]. The decision between apoptosis and other types of PCD mechanisms is known to involve caspase-8, which acts as a molecular switch for apoptosis, necroptosis, and pyroptosis [34]. Active caspase-8 blocks necroptosis and pyroptosis and rather directs cells toward apoptosis. Involvement of these alternative cell death pathways in carcinogenesis is emerging as an important field, allowing better interpretation and design of new therapeutic strategies for apoptosis-resistant cancer cells. Given their yet unresolved roles in cancer, further molecular characterization of necroptosis, pyroptosis, and ferroptosis, along with their links to apoptosis, will be of utmost importance for future anticancer strategies.

## 3. Hallmark of Cancer: Evasion of Apoptosis

Apoptosis tightly regulates tumor formation, as well as response of tumor cells to currently available treatment strategies. However, most cancer cells possess an intrinsic resistance to apoptosis and find alternative strategies to evade cell death [3,4].

Changes in the balance of pro- and antiapoptotic signal mediators, as well as mutations in key genes of apoptotic signaling pathway, are possible mechanisms underlying this resistance phenomenon. The balance of pro- and antiapoptotic signal mediators is regulated both transcriptionally (e.g., DNA hyper/hypomethylation) and post-translationally (e.g., phosphorylation) (Figure 2b). As an example of post-translational regulation, the activity of caspases is reduced in cancer cells through inactivation/degradation of proapoptotic Bid, Bim, Puma, Bad, Noxa, Bax, and Apaf-1 proteins by phosphorylation at distinct residues [4]. Overexpression of antiapoptotic proteins such as IAPs, Bcl-2, Bcl-XL and Akt or enhancement of their activity by phosphorylation can also contribute to apoptotic resistance in cancer cells [4,35]. Overexpression of cFLIP prevents clustering of FADD and caspase-8 to form DISC and consequently results in the activation of the noncanonical TRAIL pathway. The noncanonical TRAIL pathway supports survival and proliferation of cells through activation of several signaling pathways, such as PI3K/Akt, MAPK, p38, Erk, PKC, Src, IkB/NF-κB, and RIP1. Furthermore, NF-κB can induce the expression of genes encoding antiapoptotic proteins cFLIP, Bcl-XL, Mcl-1, and cIAP and, thus, contribute to apoptosis resistance [36]. On the other hand, silencing of cFLIP sensitizes cells to apoptosis [37]. Similarly, loss of proapoptotic Bax, Bak, and caspase-8 results in reduced sensitivity to apoptosis. Mutations, altered glycosylation, misregulated endocytosis, and reduced expression of death receptors, as well as overexpression of decoy receptors, may also confer apoptosis resistance. For example, DR5 mutations are identified in head and neck, breast, and lung cancer, and decoy receptor expression is elevated in TRAIL-resistant human osteoblasts [38].

## 4. Epigenetics Mechanisms of Cancer Apoptosis Evasion

Epigenetics involves the heritable modification [39] of histones and DNA, thereby modulating gene expression without altering the genetic code. However, epigenetic changes cannot be easily transmitted from one generation to the next. For transmission to occur, these epigenetic changes would have to occur in the germ cells and should escape through extensive reshaping during germ cell differentiation and the development of totipotent cells after fertilization [40]. Long-lived RNA molecules are also more likely to carry epigenetic information across generations [41], although the mechanisms remain unresolved.

DNA methylation, histone modifications, and chromatin remodeling are major epigenetic alterations that have broad effects on cell phenotype. Mammalian cells pack their long DNA (around 2 m long) into ordered structures, called chromatin, which are composed of nucleosomes. Nucleosomes consist of 146 bp DNA wrapped around octamers of DNA packaging proteins, called histones. Octamers consist of two copies of each four types of core histones (H2A, H2B, H3, and H4). Linker histone H1 is responsible for further folding and condensation of nucleosome chains to higher-ordered structures [42].

**Figure 3:**
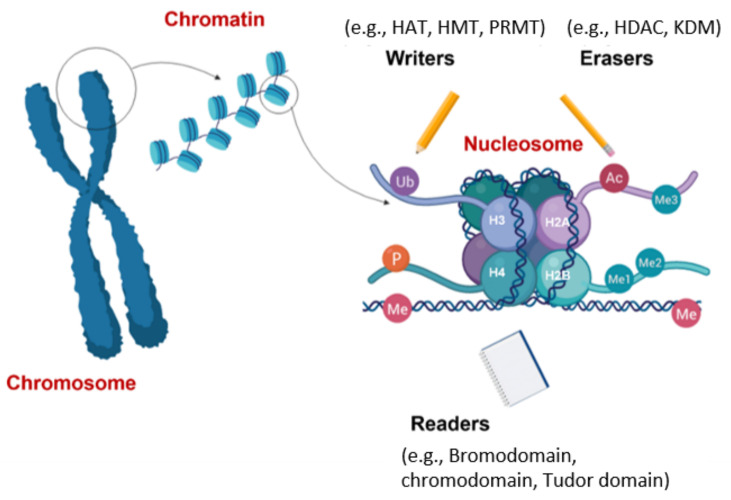
**Subgroups of chromatin modifier proteins: writers, erasers, and readers**. Post-translational marks are added by “writers”, recognized and further processed by “readers”, and removed by “erasers”. Figure generated at Biorender.com accessed on 20 April 2021.

Histone proteins are prone to a variety of post-translational modifications at their N-terminal tails, as well as at their globular core region [43]. These modifications either modulate the affinity of histones for DNA or form new binding sites for protein modules, leading to euchromatin (relaxed, actively transcribed) or heterochromatin (condensed, inactive) formation. Phosphorylation of serine or threonine, acetylation of lysine, methylation of lysine or arginine, ubiquitination of lysine, SUMOylation, carbonylation, ADP-ribosylation, and citrulation are major post-translational modifications of histones that occur dynamically [44]. These modifications (marks) are added or removed by unique chromatin-remodeling proteins, as illustrated in Figure 3. Histone-modifying enzymes that add these post-translational marks are named “writers”, enzymes that remove these modifications are called “erasers”, and proteins that recognize these marks for further regulation are called “readers” [6]. DNA methyltransferases (DNMT), histone methyltransferases (HMT), and histone acetyltransferases (HAT) are the most common writer proteins, whereas histone demethylases (HDM) and histone deacetylases (HDAC) are common erasers. Histone methylation can be associated with both transcriptional repression (e.g., *H3K27me3*, *H3K9me2* and *H3K9me3,* and *H4K20me3)* and activation (e.g., *H3K4me3*) depending on the position of the methyl group and methylation level. However, histone acetylation (e.g., *H3K9ac, H4K5ac, H4K8ac, H4K12ac*, and *H4K16ac*) leads to a euchromatin state. Post-translational modifications are recognized and further processed by various domains of epigenetic readers (namely, Bromo, Chromo, PHD, Tudor, MBT, BRCT, and PWWP domains) to regulate cellular processes, such as gene transcription, DNA repair, replication, and chromosome condensation [45].

**Figure 4:**
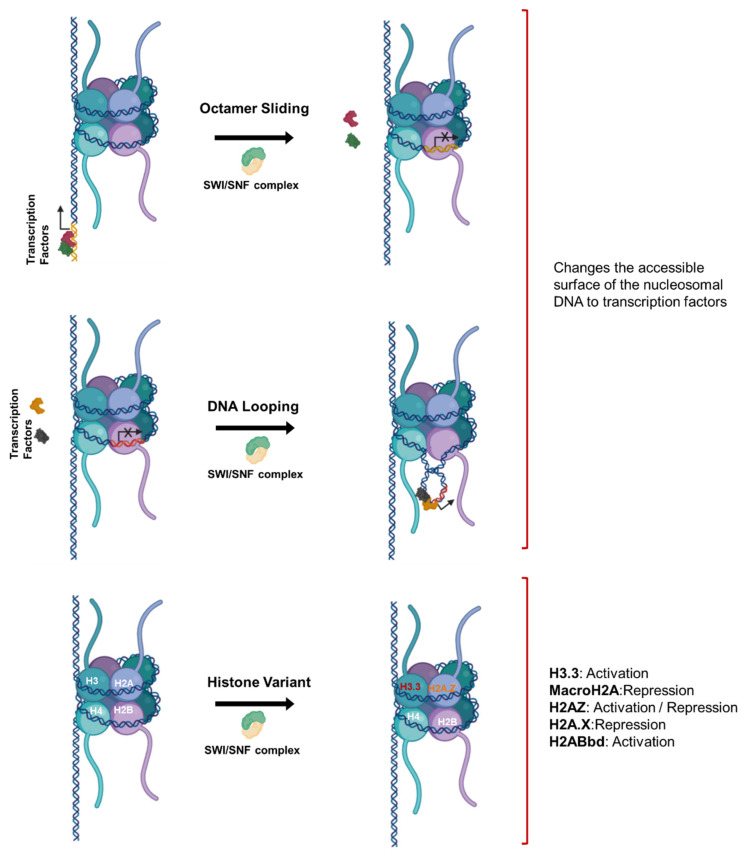
**Epigenetic modulation of gene expression through chromatin remodeling**. Octamer sliding and DNA looping change the accessible surface area of nucleosomal DNA and regulate the access of transcription factors. Histone variants have distinct functions that could activate or repress gene expression in a context-dependent manner. Figure generated at Biorender.com accessed on 20 April 2021.

Chromatin remodeling is another epigenetic alteration conducted by remodeling complexes called SWI2/SNF2, which physically modulate nucleosomes through octamer sliding, DNA looping [46,47,48], and histone substitution [49] to induce transcription of various genes, as summarized in Figure 4. Octamer sliding and DNA looping modulate the transcriptome by changing the accessible surface area of the nucleosome. The replacement of histones with their variants has distinct consequences depending on the variant type. Variants H3.3 and H2ABbd are associated with transcription activation. Replacement of H3.3 by remodeling complexes results in the immediate activation of genes previously silenced by histone H3 lysine 9 methylation [50]. On the other hand, MacroH2A and H2A.X variants repress transcription through inhibiting the binding of transcription factors. Replacement of the histone H2A with its variant H2A.Z is important for the regulation of gene expression both positively and negatively through changing the accessible surface of the nucleosome [51].

Modifications of histones modulate the capacity of genome to store and inherit genetic information and significantly differ among healthy versus tumor cells. In addition to genetic abnormalities, epigenetic alterations have major roles in initiation and progression of tumors, as well as their drug resistance. Abnormal DNA methylation and distinct histone modification patterns due to aberrant activity of epigenetic modifiers are very common in tumor cells and have effects on drug response and tumor growth [5,52].

Epigenetic functional classification divides cancer genes into epigenetic modifiers, mediators, and modulators. Epigenetic modifiers (e.g., Smarca4, PBRM1, ARID1A, ARID2, ARID1B, DNMT3A, TET2, MLL1/2/3, NSD1/2, SETD2, EZH2, and BRD4) modify the epigenome directly through DNA methylation, the post-translational modification of histones, or the alteration of the structure of chromatin, and they are frequently subjected to mutations in cancer. Emerging evidence for the role of DNMTs in malignant transformation and contribution of DNMT overexpression, mutation, and deletion to tumorigenesis was previously summarized by Zhang et al. [53]. In addition to DNA methylation, mutations in histone lysine methyltransferases SETD2 and MLL2 are frequently observed in enteropathy-associated T-cell lymphoma (93%) and follicular lymphomas (89%), acting as a driver of carcinogenesis [54,55]. SETD2-inactivating mutations are associated with poor outcome and disease relapse in renal cell carcinoma [56] and pediatric acute lymphoblastic leukemia [57]. Another HMT responsible for H3K27 methylation, EZH2, is bifunctional as either tumor-suppressing or tumor-promoting depending on the cancer type. EZH2 is subjected to gain-of-function mutations and is overexpressed in various solid tumors (breast, ovarian, lung, liver, bladder, glioblastoma), whereas it is inactivated through deletions and frameshift, nonsense, and missense mutations in myeloproliferative neoplasms (MPNs) and in human T-cell acute lymphoblastic leukemia [58]. Histone acetylation is as important as methylation for initiation of carcinogenesis. Mutations and translocations in HAT family members (p300, CBP, and MYSTA4) have been observed in both hematological malignancies and solid tumors. In line with this evidence, massive loss of histone acetylation through germline mutations and overexpression of HDACs silence tumor suppressor genes in various tumor types [59]. Mutations in enhancers or transcription factor-binding sites, which prevent the binding of chromatin regulators, are also drivers of oncogenesis and can be classified as epigenetic modifiers. DNA and histone demethylation also contribute to the resistance to apoptosis. K370me2 demethylation by KDM1A suppresses the apoptosis initiator function of p53 by blocking its interaction with 53BP1 [60]. In line with this evidence, KDM1A inhibitor, HCI-2509, triggered apoptosis in Ewing sarcoma cell lines, endometrial carcinoma, and AML cells as a single agent, as well as in combination with anticancer drugs [61,62,63]. Another potent KDM1A inhibitor, GSK2879552, is under Phase I clinical trial (*NCT02177812*) for patients with relapsed/refractory AML. KDM3A was illustrated to facilitate tumor growth [64], modulate invasion and apoptosis of breast tumor cells by targeting histones and p53 [65], and sustain myeloma cell survival [66]. Another member of the KDM family, KDM6B, has context-dependent function in tumorigenesis. KDM6B elevates the expression of antiapoptotic protein Bcl-2 in an ERα-dependent fashion in breast tumors [67]. Oppositely, KDM6B prevents carcinogenesis by stabilizing nuclear p53 and results in partial cell-cycle arrest and differentiation in germline stem cells [68]. TET family DNA demethylases also modulate the apoptotic response of tumor cells. TET2 mutations affect non-CpG island DNA methylation at enhancers and transcription factor-binding sites in chronic myelomonocytic leukemia [69]. TET1 expression regulates proliferation and apoptosis in osteosarcoma cells [70]. Vitamin C promotes apoptosis in breast cancer cells by increasing TRAIL expression through TET-dependent DNA demethylation [71].

On the other hand, epigenetic mediators (OCT4, NANOG, LIN28, SOX2, and KLF4) are targets of epigenetic modifications and are responsible for the emergence of cancer stem cells (CSCs). ncRNAs are also epigenetic mediators associated with carcinogenesis. Misregulation of ncRNA expression through amplifications, deletions, mutations, and epigenetic modulation is also associated with tumorigenesis. DNA methylation-mediated silencing of miR-34b/c, miR-148, and miR-93 results in aberrant activity of oncogenic C-MYC, E2F3, CDK6, and TGIF2 and promotes invasiveness and metastasis of tumor cells [72]. Lastly, epigenetic modulators (IDH1/2, KRAS, APC, TP53, STAT1/3, YAP1, and CTCF) lie upstream of the modifiers and mediators in signaling. These modulators relay signals from environmental stress, injury, inflammation, and aging to modifiers to epigenetically alter tumor suppressors or oncogenes [73]. Heterozygous somatic mutations in IDH1/2 are evident in ~20% of AMLs and are linked to global hypermethylation, as well as gene-specific methylation signatures [74].

Several epigenetic mechanisms are misregulated in cancer cells, and epigenetic regulation of apoptosis can be categorized into three major themes as summarized below: evading apoptosis (1) by aberrant DNA methylation, (2) by aberrant histone modifications, and (3) by epigenetic regulation of miRNAs.

### 4.1. Evading Apoptosis by Aberrant DNA Methylation

**Figure 5:**
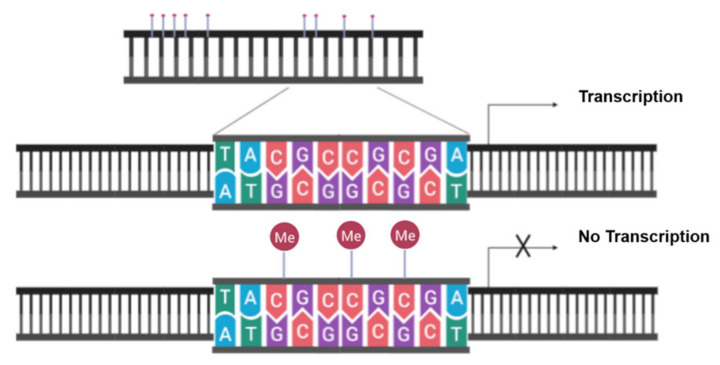
**CpG island methylation blocks transcription.** Methylation blocks the access of transcription factors to CpG sites and, therefore, results in transcriptional silencing. Figure generated at Biorender.com accessed on 20 April 2021.

Regions in DNA where a C nucleotide is followed by a G nucleotide in the 5′→3′ direction are called CpG sites. CpG nucleotide-rich regions (300–3000 bp) in mammalian genomes are called CpG islands, and these islands reside in very close proximity to gene promoters [75]. The methyl residue is transferred from a methyl donor SAM (*S*-adenosyl-l-methionine) to the 5′ carbon of cytosine in CpG sites by enzymes called DNA methyltransferases (DNMTs). DNMT1 functions as a ‘maintenance’ methyltransferase and is responsible for copying DNA methylation during cell division, while DNMT3A and DNMT3B are de novo methyltransferases, which do not require a methylated template [76,77]. The methyl motif attracts specific methyl-DNA-binding proteins (e.g., MeCP2, MBD1, MBD2, MBD3, MBD4, and KAISO) and blocks the access of transcription factors (e.g., AP-2, c-Myc/Myn, CREB, E2F, and NF-κB) to CpG sites, resulting in transcriptional silencing, as illustrated in Figure 5 [78]. Therefore, CpG island methylations at promoter sites are tightly regulated.

CpG islands at tumor suppressor and proapoptotic gene promoters are mostly hypermethylated in cancer cells due to DNMT overexpression [79] or gene mutations (e.g., *IDH1* [80], *SDH* [81], *TET2* [69]), which lead to uncontrolled division and growth of cells [82]. Hypermethylation blocks the initiation and progression of both intrinsic and extrinsic apoptosis by modulating the expression of major players of cell death cascade, as previously reviewed by Elmallah and Micheau [83]. Promoter hypermethylation-mediated silencing of *FAS* expression renders colonic epithelium cells resistant to apoptosis and contributes to neoplastic transformation into cutaneous T-cell lymphoma [84] or colon carcinoma [85]. Similarly, *DR4* and *DR5* promoter methylation results in resistance to TRAIL-induced apoptosis in human neuroblastoma [86], melanoma [87], and ovarian cancer [88]. Caspase-8/10 silencing by promoter methylation disrupts the cycle of apoptosis in hepatocellular carcinoma [89], bladder cancer [90], small-cell lung carcinoma [91], glioblastoma [92], retinoblastoma, and neuroblastoma [93]. In renal cell carcinoma and chronic myeloid leukemia, silencing of *BIM* by promoter hypermethylation confers resistance to potent apoptosis induction [94]. *APAF*-1 silencing is evident in leukemia [95] and melanoma [96], as well as gastric [97], bladder, and kidney cancer [98], and it is correlated with therapy resistance. In gastric and bladder cancer, XAF1, an inhibitor of antiapoptotic XIAP, is downregulated by promoter hypermethylation, and this is correlated with pathology and clinical outcome [99,100,101]. *BCL*-2 promoter is hypermethylated in prostate cancer [102]. Proapoptotic *BAX*, *BAK* and *PUMA* genes are also subject to promoter hypermethylation-mediated silencing in multiple myeloma cells [103] and Burkitt’s lymphoma [104]. Proapoptotic *BAD* promoter is hypermethylated in myeloma [103]. *BCL*-*2L10* hypermethylation is observed in gastric cancer [105] and leukemia [106]. *BIK* is downregulated by hypermethylation in glioma [107], RCC [108], prostate cancer [109], and myeloma [110]. *BNIP3* levels are modulated through methylation for gastric cancer [111], colorectal cancer [112], leukemia [113], and HCC [114]. Proapoptotic *HRK* hypermethylation is evident in colorectal, gastric [115], glioblastoma [116], PCNSL [117], and prostate cancer [118].

Other critical genes affected by hypermethylation are *p16^INK4a^*, a cell-cycle inhibitor; *APC*, a cell cycle regulator; and *MGMT*, *BRCA1*, and *MLH1,* DNA repair genes [119]. Silencing of the *MGMT* gene by promoter hypermethylation predicts patient response to temozolomide treatment and is associated with better prognosis in glioma [120]. Tumor suppressor *DAPK* (death-associated protein kinase) was previously shown to be silenced by promoter hypermethylation in colorectal [121] and lung cancers [122] and B-cell lymphoma [123], which renders cancer cells less responsive to TNF-induced apoptosis. *HIC1* (hypermethylated in cancer 1) gene expression is found in various cancers to be silenced by DNA hypermethylation [124,125]. The loss of HIC1 results in inactivation of p53, allowing cells to bypass apoptosis and survive DNA damage. The *TP53* gene itself is also subjected to hypermethylation in acute lymphatic leukemia patients [126]. *RASSF1, a* tumor suppressor gene, is also hypermethylated in several human tumors, such as parathyroid tumors [127], nasopharyngeal carcinoma [128], non-small-cell lung cancer [129], and hepatoblastoma [130].

In addition to gene silencing, CpG island methylation can cause destabilizing of genetic mutations and consequent tumorigenesis due to the favorable conversion to thymine by hydrolysis of the amine group. CpG islands are sites to ~35% of all point mutations in the germline [131] and important hotspots for acquired somatic mutations leading to cancer [132,133]. In addition, cytosine methylations modulate the UV light absorption level of the nucleotide base, creating pyrimidine dimers [134].

**Figure 6:**
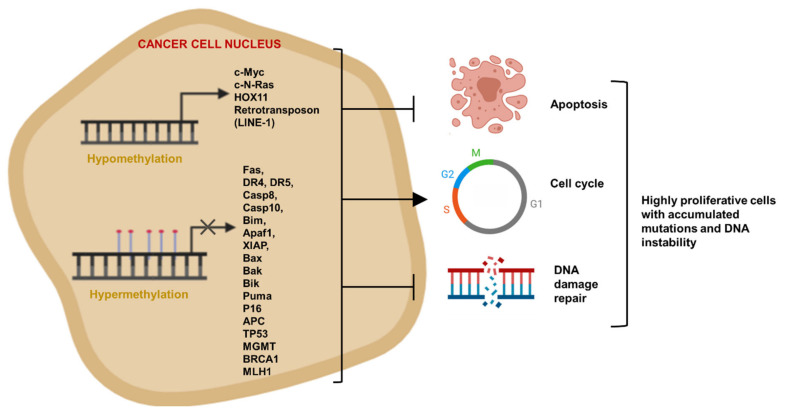
**Evasion of apoptosis by DNA hypermethylation and global hypomethylation.** CpG islands at promoters of tumor suppressor, proapoptotic, cell-cycle regulator, and DNA damage repair genes are mostly hypermethylated in cancer cells due to DNMT overexpression or gene mutations, which lead to uncontrolled division and growth of cells. Cancer-associated hypomethylation occurs in several tumor-initiator or proliferation-associated genes and leads to chromosome instability. Figure generated at Biorender.com accessed on 20 April 2021.

In addition to aberrant hypermethylation, cancer cells possess global genomic hypomethylation, which contributes to their highly proliferative and apoptosis resistant phenotype. Cancer-associated DNA hypomethylation in the human genome was discovered in 1983 [135,136]; yet, at the time, the biological significance of this finding was not clearly understood. Global genomic hypomethylation is evident in various types of human cancer, including prostate metastatic tumors [137], B-cell chronic lymphocytic leukemia [138], hepatocellular carcinomas [139], and cervical cancer [140], as well as colorectal cancer, gastric cancer, and melanomas [5]. Heterochromatin repeats (e.g., satellite DNA, endogenous retrotransposons) are shown to be hypomethylated in various cancer types, and such a pattern could affect chromatin structure and genomic stability, in addition to possible effects on transcription of other parts of the genome. Hypomethylation can lead to chromosome instability through translocations and deletions due to loosening of chromatin structure, reactivation of transposable elements, and mitotic recombination [141]. Interspersed repeat (e.g., retrotransposon LINE-1) hypomethylation is observed in chronic lymphocytic leukemia [142], urinary bladder carcinomas [143], hepatocellular carcinomas [144], and prostate carcinomas [145]. Oncogene promoters and some gene regulatory sequences are mostly hypomethylated in cancer cells [146]. Cancer-associated hypomethylation occurs also in several tumor-initiator or proliferation-associated genes, such as *pS2*gene [147], *HOX11* proto-oncogene [148], and c-*MYC* and *c-N-ras* oncogenes [149,150]. Contribution of aberrant DNA methylation to apoptosis resistance is summarized in Figure 6.

### 4.2. Evasion of Apoptosis by Aberrant Histone Modifications

CpG island hypermethylation commonly observed in cancer cells is interconnected with histone marks, such as elevated H3K9me and H3K27me3 levels, as well as H3 and H4 deacetylation and loss of H3K4me3 [151,152,153]. The relationship between DNA methylation and histone modifications is established with the help of methyl-DNA binding proteins, such as MeCP2, MBD1, and Kaiso [154]. These proteins interact with methylated CpG islands and recruit histone deacetylases (HDACs, Sirtuins) and histone methyltransferase complexes to the site. Similarly, the histone code can further determine DNA methylation patterns by recruiting methyltransferase enzymes. For instance, histone methyltransferase G9a recruits DNA methyltransferases, DNMT3A and DNMT3b, to promoter sites [155].

Aberrant histone modifications are among hallmarks of cancer. Some of the histone marks related to tumorigenesis are reduced acetylation of H3 and H4 due to high HDAC or low HAT activity, decreased H3K4me3 mark, and increased H3K9me3 and H3K27me3 modifications, which can silence tumor suppressor genes or proapoptotic genes to facilitate uncontrolled growth of cells. Deprivation of H4K16ac and H4K20me3 has been suggested to be a prevalent hallmark of cancer cells due to their contribution to chromosome instability [156]. Phosphorylation of histone H2A, H2B, H3, and H4, dephosphorylation of histone H1, and deubiquitylation of histone H2A have also been linked to the apoptotic process [157]. Cancer cells adopt epigenetic mechanisms, such as increasing the H3K4me3 permissive mark to increase expression of DNA repair genes such as *BRCA1*, *BRCA2*, or *MGMT* and consequently avoid drug toxicity [35]. Hypoxic stress-mediated epigenetic silencing of the DNA mismatch repair gene, *MLH1,* is established by decreased H3K4 methylation at the promoter via demethylases [158]. Other apoptosis-related genes repressed by epigenetic mechanisms include *p16^INK4a^* [159], *p57^Kip2^* [160], *GAS2*, *PIK3CG*, and *p21^Waf^* [161].

Separately, several histone modifications are involved in chromatin alterations such as inter-nucleosomal DNA fragmentation, chromatin condensation, and increased chromatin accessibility, which are all related to apoptosis. To this end, protein complexes governing cell death and survival decisions might be recruited by specific epigenetic histone marks. For instance, H2A.X-Y142 phosphorylation inhibits MDC1-mediated binding of DNA repair factors (MRE11, RAD50, NBS1, 53BP1, and BRCA1) to H2A.X-S139ph (*γ*-H2A.X) sites, instead facilitating the recruitment of proapoptotic components, thus modulating cell fate after DNA damage induction [162,163]. The H2B-S14ph mark has been linked to chromatin condensation [164] and inter-nucleosomal DNA fragmentation [165], in addition to its contribution to the inhibition of survival related genes, such as *NF*-*κB* [166]. Similarly, PKC*δ-*mediated phosphorylation of H3T45 induces structural changes within the nucleosome and augments DNA fragmentation during late apoptosis [167]. Therefore, harmonious coordination of a wide variety of histone marks is critical for the determination of cell fate.

In addition to histone marks, histone variants by themselves can contribute to tumorigenesis. For example, high levels of histone variant H2A.Z are evident in several cancer types, such as hepatocellular carcinoma and bladder cancer. This contributes to proliferation and genomic instability and reshaping of the chromatin structure so that new genes get activated through recruitment of transcription machinery [168]. H2A.Z knockdown was shown to downregulate *BCL*-2 and upregulate *BAK*, *CASPASE*-3, and *CASPASE*-9 in in intrahepatic cholangiocarcinoma [169]. Components of the apoptosis pathway can also be subjected to direct regulation by aberrant histone modifications. *DR4* gene expression is modulated by aberrant H3 and H4 acetylation patterns at the promoter site in medulloblastoma patients [170]. Similarly, abnormal H3 and H4 acetylation patterns modulate proapoptotic Bax protein levels [171]. The H3K27me3 repressive mark modulates proapoptotic *BIM* levels in Burkitt’s lymphoma [172].

In addition to their roles in gene regulation through histone modifications, HATs and HDACs can contribute to tumorigenesis by modifying non-histone proteins such as Rb, E2F, p53, ku70, and TFIIF [173]. For example, acetylation-dependent stabilization elevates E2F1-mediated apoptosis upon genotoxic stress [174]. Another example is the regulation of Ku70, whose expression is induced upon DNA damage playing a critical role in apoptosis regulation. Ku70 normally interferes with Bax activation through blocking its translocation to mitochondrial membrane and inhibiting apoptosis. Two important lysine residues of Ku70 (lysines 539 and 542) are acetylated by CBP and PCAF, which disrupts the Ku70–Bax interaction and, thus, facilitates apoptosis [175].

**Figure 7:**
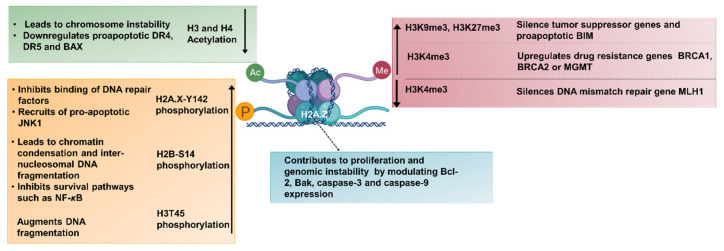
**Aberrant histone modifications and their contribution to evasion of apoptosis.** Histone marks related to Table 3. and H4 due to high HDAC or low HAT activity, decreased H3K4me3 mark, and increased H3K9me3 and H3K27me3 modifications, which can silence tumor suppressor genes or proapoptotic genes to facilitate uncontrolled proliferation of cells. Histone phosphorylation and incorporation of histone variants also contribute to tumorigenesis by promoting proliferation and genomic instability. Figure generated at Biorender.com accessed on 20 April 2021.

Aberrant histone modifications and their contribution to evasion of apoptosis are summarized in Figure 7.

### 4.3. Evading Apoptosis by Epigenetic Regulation of miRNAs

**Figure 8:**
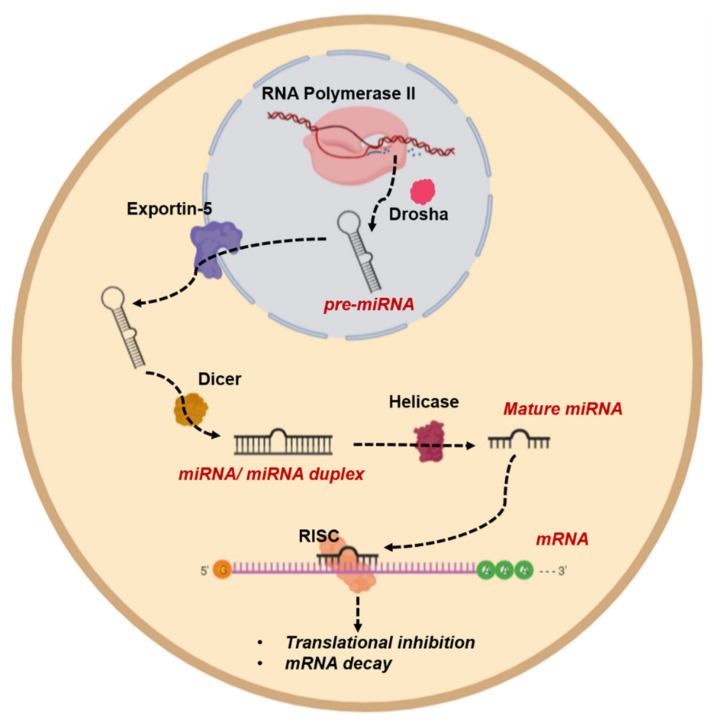
**miRNA generation and action in the cell.** miRNAs are small noncoding RNAs endogenously expressed in the cell and generated through a multistep process. RNA polymerase II synthe Table 5. pre-miRNAs are transported to the cytoplasm and get cleaved in the cytoplasm by Dicer into mature miRNA duplexes. Upon separation of the duplex, the guide strand gets loaded into the RISC complex and scans the transcriptome for complementary sites. Upon binding to complementary mRNA, RISC initiates either mRNA degradation or translation repression to control gene expression. Figure generated at Biorender.com accessed on 20 April 2021.

Aberrant DNA methylations and histone modifications also affect microRNA (miRNA) expression in cancer cells. miRNAs are small noncoding RNAs endogenously expressed in the cells; they are responsible for the regulation of gene expression and have broad effects on proliferation, differentiation, and apoptosis [176]. As illustrated in Figure 8, miRNA generation is a multistep process. It starts in the nucleus through RNA polymerase II-mediated synthesis of hairpin-structured primary miRNAs (pri-miRNAs), which are further processed by nuclear endonuclease Drosha into 70 nt long precursor miRNAs (pre-miRNAs). Pre-miRNAs are transported to the cytoplasm by Exportin-5 through the nuclear pore complex and get cleaved in the cytoplasm by Dicer into mature 21–23 nt long miRNA duplexes. Upon separation of the duplex, the guide strand gets loaded into the RISC complex and scans the transcriptome for complementary sites. Upon binding to complementary mRNA, RISC initiates either mRNA degradation or translation repression to control gene expression [177].

Some miRNAs with critical gene targets involved in apoptosis can be modulated by aberrant DNA and histone modifications in cancer cells. Some examples are described below. miR15/16 miRNAs silence antiapoptotic *BCL*-2, as well as *CYCLIND1*, *MCL1*, and *WNT3A* at the post-transcriptional level [178,179]. In several human malignancies such as pituitary adenoma [180] and B-cell chronic lymphocyte leukemia [181], downregulation of the miR-15/16 cluster is evident. The miR15/16 cluster is epigenetically silenced by histone deacetylation [181]. Indeed, the Myc protein represses miR-15a/16-1 cluster expression through recruitment of HDAC3 in mantle cell lymphoma [182]. Another example is the miR-34 family, which can be directly induced by p53 and can target *CYCLINE2, MET, MYCN, NOTCH1/2, CDK4/6*, and antiapoptotic *BCL*-2 [183]. Similarly, miR-34 is repressed via hypermethylation in human gastric cancer, chronic lymphocytic leukemia, pancreatic, breast, colon, and kidney cancer, and Burkitt’s lymphoma [184,185]. Another example is miR-29b, which targets *DNMT3b* [186] and *MCL*1 and is remarkably downregulated in lung [187], prostate [188], bladder [189], and ovarian cancers [190], as well as glioblastomas [191]. miR-193a-3p, miR-512-5p, miR-153, and miR-133B also target *MCL1* and are repressed via hypermethylation in AML [192], gastric tumors [193], glioblastoma [194], and lung cancer [195], respectively. DNMT inhibitor 5-aza-2′-deoxycytidine and HDAC inhibitor 4-phenylbutyric acid can restore the expression of the silenced miR-512-5p in human gastric cancer cells [193], attesting to its epigenetic regulation. Similarly, miR-127 that directly targets proto-oncogene *BCL-6* [196] is hypermethylated in bladder, prostate, breast, and lung cancer, as well as lymphoma [197]. Two other examples are miR-106b and miR-93, which are known to impair TGFβ-induced apoptosis through inhibition of *BIM* expression in gastric cancer cells [198]. miR-106b and miR-93 are intronic miRNAs, and their transcription is modulated by the CpG islands located in the promoter of the host gene MCM7. SAHA, an HDAC inhibitor, can repress their expression by repressing MCM7 in hepatocellular carcinoma cells [199].

There are several other cancer-associated miRNAs whose expression modulation is not very well understood from the epigenetics aspect. For example, the miR-221 and miR-222 cluster targets several genes including *PTEN*, *TIMP3*, *p27^Kip1^*, *p57^Kip2^*, *DDIT4*, *FOXO3A* [200], and proapoptotic *PUMA* [201] and *CASPASE-3* [202], and it is upregulated in multiple solid tumors such as bladder cancer [203] and glioma [204]. The miR-17-92 cluster and its paralog miR-106b-93-25 cluster target *p21^Cip1^* and proapoptotic *BIM* [205], and they are known to be overexpressed in multiple solid tumors, including lung and colon cancer, lymphoma, medulloblastoma, and multiple myeloma [206,207]. miR-135a inhibits *JAK2* and results in consequent downregulation of antiapoptotic Bcl-xL [208,209]. miR-135a is downregulated in classic Hodgkin lymphoma, AML [210], and ovarian cancer [211]. miR-491 also targets *BCL*-xL in colorectal cancer [212]. Egr2 is a tumor-suppressive transcription factor, which induces apoptosis through Bnip3L and Bak activation [213]. miR-150 targets *EGR2* to promote gastric cancer progression [214]. Macrophage migration inhibitory factor (MIF) triggers apoptosis in gastric epithelial cells through repressing p53 phosphorylation and upregulating *BCL-2* expression [215]. miR-451 targets MIF and is downregulated in gastric cancer [216]. Epstein–Barr virus (EBV) is the first human virus discovered to express miRNA called miR-BART5 [217], which is associated with gastric cancer [218]. miR-BART5 renders gastric cancer cells resistant to apoptosis by targeting *PUMA* [219]. PTEN is a tumor suppressor that facilitates apoptosis through negatively regulating the PI3K/Akt survival pathway [220]. *PTEN* is targeted by miR-21 [221], whose expression is elevated in gastric cancer tissues. miR-375 also modulates the activity of PI3K/Akt pathway through direct targeting of *PDK1* and is downregulated in gastric cancer [222]. NF-κB signaling is also inhibitor of apoptosis [223] and is directly targeted by miR-9, which is downregulated in gastric cancer.

Epigenetic strategies of apoptosis evasion adopted by tumor cells are summarized in Table 1, Table 2 and Table 3 by extending previous the review of Hajji and Joseph [78].

## 5. Reprogramming the Cancer Epigenome by Epigenetic Drugs to Trigger Tumor Cell Death

Given the importance of epigenetic changes in cancer, reversion of DNA and histone modifications by epigenetic drug (Epi-drug) interventions can provide a therapeutic advantage for various type of cancers. To this end, several drugs that target the epigenetic landscape of tumor cells have been developed and received clinical attention. Some of these “Epi-drugs” and their implications are summarized below.

DNMT1 inhibitors result in gradual hypomethylation across cell divisions and lead to elevated expression of tumor suppressor genes. Azacitidine, decitabine, guadecitabine, and 4-thio-2-deoxycytidine are DNMT1 inhibitors designed for clinical use [224,225]. Azacitidine and its deoxy derivative decitabine are approved by the US Food and Drug Administration (FDA) for treatment of myelodysplastic syndromes (MDS) and AML since they decrease malignant cell burden and improve blood cell count and patient survival [226,227]. Guadecitabine has a longer effective half-life due to its improved pharmacology and pharmacodynamics, and it has shown promise in early clinical trials [228]. 4-Thio-2-deoxycytidine is orally bioavailable and is currently in a Phase I trial in patients with advanced solid tumors [224]. Separately, mutated forms of isocitrate dehydrogenase *(IDH1* and *IDH2*) genes are known to be associated with aberrant DNA methylation in cancer. To this end, IDH1 and IDH2 inhibitors, AG-120, AG-221, AG-881, and IDH305, are currently in clinical trials for low-grade glioma and AML patients.

HDAC inhibitors constitute the largest group of epigenetic drugs and are known to exert their activity through maintaining the expression of tumor suppressor genes. Vorinostat (SAHA) [229,230], belinostat [231], and romidepsin [232,233] are FDA-approved for treatment of cutaneous or peripheral T-cell lymphoma. Panobinostat is approved for treatment of drug-resistant multiple myeloma in combination with the proteasome inhibitor bortezomib [234].

Histone acetylation readers, i.e., the bromodomain and extra-terminal (BET) proteins, are also pharmacological targets for cancer therapy. Of these, BRD4 inhibitor OTX015 mediates a rapid tumor regression with low toxicity [235]. Other BET inhibitors, such as ABBV-075, BMS-986158, and GSK2820151, are also in clinical trials for several malignancies.

The histone methyltransferase EZH2 generates the H3K27me3 mark, which leads to transcriptional repression [236]. EZH2 inhibitors, such as tazemetostat, CPI-1205, DS-3201, and GSK2816126, are in clinical trials. Inhibition of EZH2 blocks proliferation of the drug-resistant stem-cell population, thereby preventing tumor growth [237]. Pinometostat, an inhibitor of H3K79 methyltransferase DOT1L, recently completed a Phase I clinical trial in AML patients. The demethylase LSD1 gene has been shown to play an important role in cancer and is very highly expressed in several cancer cell lines [63,238,239]. LSD1 inhibitors GSK2879552 and INCB059872 are in clinical trials for patients with AML, MDS, and small-cell lung cancer.

As another approach, miRNAs have high potential as therapeutic tools against cancer due to their ability to regulate multiple targets. Therapeutic strategies focus mainly on either reactivation of tumor suppressor genes through inhibition of oncogenic miRNAs or inhibition of oncogenic gene activity by supplementing cells with miRNA mimics. miRNA inhibitors includes antisense anti-miR oligonucleotides (AMOs, antagomirs) [240], locked nucleic acid (LNA) [241], miRNA sponges [242], and small-molecule inhibitors of miRNAs (SMIR) [243]. To exemplify, LNA silencing of miR-21 reduced cell viability through increasing intracellular caspases activity in glioblastomas [244]. An miR-15a mimic triggered apoptosis and prevented proliferation of prostate cancer cell lines [179]. The Let-7 miRNA precursor blocked proliferation of tumors in K-ras mutant mouse [245,246]. The miR-34 mimic MRX34 resulted in tumor regression in liver cancer orthotopic mouse models [247]. In a Phase I clinical trial (*NCT01829971*), MRX34 showed strong activity in hepatocellular carcinoma, renal cell carcinoma, and melanoma through repression of target oncogenes namely *FOXP1, BCL2, HDAC1*, and *CTNNB1* [248]. However, the trial was halted due to immunological adverse effects. Intravenous administration of tumor suppressive miR-16 mimic was under a Phase I clinical (*NCT02369198)* for advanced non-small-cell lung cancer (NSCLC) and malignant mesothelioma (MPM), whereby efficacy and good tolerability were reported. MRG-106, a synthetic microRNA antagonist of miR-155, is currently under a Phase II trial for patients with cutaneous T-cell lymphoma (*NCT03713320*). In addition, DNMT inhibitor 5-aza-2′-deoxycytidine and HDAC inhibitor 4-phenylbutyric acid were shown to induce miR512-5p expression in human gastric cancer to trigger apoptosis by suppressing the Mcl-1 [193]. HDAC inhibitors vorinostat and trichostatin A (TSA) decrease proliferation and increase apoptosis in colorectal cancer by downregulating miR-17-92 cluster expression, consequently elevating *PTEN*, *BCL-2L11*, and *CDKN1A* expression [249].

Epigenetic modulations can also be utilized to overcome drug resistance in several cancer types through a combinatorial treatment approach. Several cell culture [250,251] and in vivo models [252,253,254] evidenced the efficacy of combinatorial treatment of DNMT and HDAC inhibitors for various cancer cells. Combinatorial treatment of HDAC inhibitor TSA with the DNMT inhibitor decitabine results in reactivation of densely methylated tumor suppressor genes [250]. TSA, belinostat, and vorinostat show synergistic activity with conventional chemotherapeutic agents such as paclitaxel [255], gemcitabine [256], cisplatin [257], etoposide, and doxorubicin [258]. Decitabine acts synergistically with paclitaxel [259,260] and cisplatin [254]. Non-small-cell lung cancer cells are sensitized to EGF tyrosine kinase inhibitors (TKIs) by a HDAC inhibitor [261]. Bromodomain inhibitor JQ1 similarly sensitizes T-cell acute lymphoblastic leukemia to γ-secretase inhibitor-mediated apoptosis [262]. HDAC inhibitors can also sensitize cancer cells to ionizing radiation-mediated apoptosis by modulating cell cycle and growth-related gene expression. HDAC inhibitor sodium butyrate elevates the radiosensitivity of human colon carcinoma cell lines [263]. TSA, entinostat, valproic acid, tributyrin, vorinostat, bicyclic depsipeptide, and hydroxamic acid analogues were shown to sensitize various cancer cell lines toward ionizing radiation [264,265].

Epi-dugs currently under investigation for various malignancies are summarized in Table 4.

Modulation of death receptor-mediated pathways by Epi-drugs has been one successful approach for better apoptosis response of tumor cells. To this end, HDAC inhibitors MS275 [266], SAHA [267], valproic acid [268], depsipeptide [269], SBHA [270], and LAQ824 [271] have been shown to augment TRAIL responses in various tumor types including prostate cancer, primary myeloid leukemia, melanoma, breast cancer, medulloblastoma, glioblastoma, and CLL. HDAC-mediated sensitization involves upregulating the DR expression and proapoptotic gene activity (*BID, BAD, CASPASES, P21, BAK,* and *BAX*) and downregulating antiapoptotic proteins (e.g., Cflar, Bcl-2, Bcl-xL, Xiap, Mcl1, Survivin, and CyclinD1). MS275 elevates acetylation of H3 and H4 at the DR4 promoter and causes an increase in TRAIL receptor expression in medulloblastoma cells. Under combinational treatment with MS275, medulloblastoma cells are much more prone to TRAIL-induced cell death [170]. HDAC inhibitors can also induce expression of proapoptotic genes such as *BAX* and *BAK* while blocking the expression of antiapoptotic genes such as *XIAP* and *CFLAR*, consequently sensitizing tumor cells to extrinsic and intrinsic apoptosis [52].

On the other hand, some epigenetic changes might contribute to apoptosis resistance due to gene silencing. Modulation of DNA methylation with the methyltransferase inhibitor 5-aza-2′-deoxycytidine has been proven to be effective in modulating the TRAIL response via restored caspase-8 expression [272,273,274]. Combination of the DNMT inhibitor decitabine with valproic acid significantly increases caspase-8 expression in SCLC and sensitizes tumor cells to TRAIL [275]. DNMT1 and DNMT3b silencing was shown to sensitize human hepatoma cells via upregulation of DR5 and caspase-8 [276]. Similarly, in Burkitt’s lymphoma, DNMT1 inhibitor Iso-3 synergizes with TRAIL via a reduction in survivin expression and induction of DR5 surface expression [277]. Taken together, epigenetic modulation of apoptosis sensitivity/resistance with Epi-drugs can serve as a promising approach for cancer therapy.

There are various challenges for the implementation of epigenetic drugs in clinical use. First of all, epigenetic factors reside in large chromatin complexes and act in interplay to regulate gene expression. Therefore, targeting a single epigenetic factor might have unwanted effects on other chromatin-related proteins [278]. To exemplify, targeting histone deacetylases (by vorinostat, sodium butyrate, or TSA) can alter the methylation status, as well as chromatin remodeling, of the targeted location as in the case of the effect of HDAC inhibitors on histone methyltransferase JARID1 [279]. Therefore, in vitro genetic studies or assays for compound screening might overlook that crosstalk between epigenetic factors. In vivo screening assays with focused chemical or genetic libraries for the epigenetic enzymes can overcome this obstacle. Secondly, substrates of epigenetic enzymes are not confined to chromatin proteins, but they may include oncogenes as in the cases of p300/CBP-mediated acetylation and SMYD2-mediated methylation of p53 [280,281]. Therefore, off-target effects of the potential epigenetic compounds should be deeply considered. This off-target effect of epigenetic modifier enzymes could be minimized by sequence-specific gene modulation through DNA-targeting tools, as well as through temporal control of epigenetic modulation with the help of optogenetics. Transcriptional activator-like effectors (TALEs) and clustered regularly interspaced short palindromic repeat (CRISPR)/Cas approaches are utilized to localize epigenetic modifier enzymes to specific DNA sequences [282]. The catalytic domain of an epigenetic enzyme can be fused to dCas9 and modulate the epigenome at particular locations [283]. On the other hand, optogenetics enable temporally specific modulation of the epigenome with the help of the light-sensitive protein Cryptochrome 2 (Cry 2) [284].

Another challenge for the utilization of epigenetic drugs in clinical use is their low isoform selectivity. To exemplify, DNMT inhibitors simultaneously target DNMT1, 3A, and 3B; therefore, it is challenging to understand effects of different isoforms in tumorigenesis. Most of the time, inhibition of a single epigenetic enzyme may not possess sustained effects due to compensation of the epigenetic mark by other epigenetic modifiers. Combinational therapeutics targeting multiple epigenetic marks of the target genomic loci is, therefore, expected to result in sustained effects for many cell divisions [285,286].

Arranging the treatment period for epigenetic drugs is also challenging since epigenetic programming takes time to exert its function. On the other hand, low-dose treatments showed a superior effect without causing immediate cytotoxicity [287]. Overall, efficient transport and delivery systems, which can readily and specifically target tumor tissues, and low-dose administration without causing general toxicity will be two key strategies for effective future Epi-drug based cancer treatments.

## 6. Conclusions and Future Perspectives

Evasion of apoptosis plays a major role for the emergence and progression of a wide variety of cancers. Modulation of apoptosis-related genes via epigenetic alterations has gained increasing attention as our comprehension of the cancer epigenome has rapidly grown with the discovery of novel epigenetic modifier enzymes and their target oncogenes/tumor suppressors. Global changes of epigenome via aberrant modifications of DNA and histones, as well as altered miRNA expression, modulate the expression of genes critical to apoptosis and render malignant cells resistant to current therapies. Increased understanding of tumor-specific epigenetic alteration of apoptosis will enable the discovery of novel targeted therapies utilizing Epi-drugs. Despite the complexity and heterogeneous nature of cancer, Epi-drugs hold great promise for improved survival of patients alone or in combinatorial approach with other therapeutic modalities due to their potential of resetting the cancer epigenome. Detection of the clear pattern of epigenetic changes in early versus late stages of tumors via high-throughput, robust, and affordable methodologies will ultimately lead to rapid discovery of novel epigenetic biomarkers and production of effective Epi-drugs. Optimization of dosage and timing of Epi-drugs will enable to revert therapy resistance and to overcome side-effects which are currently main obstacles for cancer therapy. Ultimately, reprogramming the epigenome of cancer cells toward more apoptosis-mediated cell death may become possible with Epi-drugs. However, open questions in field of epigenetics (Is there a histone code copied during DNA replication in cancers? How is gene regulation controlled by noncoding elements like introns in normal versus cancer cells? How do environmental and dietary factors affect the epigenetic phenotype of tumor cells?) remain to be answered for the development of personalized and effective treatment modalities for cancer.

## Figures and Tables

**Table 1:** Epigenetic modification of the core apoptotic machinery.

Pro/Antiapoptotic Genes	Epigenetic Modification	Outcome	Cancer Type
*FAS*	DNA hypermethylation	Downregulation	T-cell lymphoma [84], colon carcinoma [85]
*DR4/DR5*	DNA hypermethylation	Downregulation	Neuroblastoma [86], melanoma [87] and ovarian cancer [88],
	H3 and H4 deacetylation	Downregulation	Medulloblastoma [170]
*CASPASE-8/10*	DNA hypermethylation	Downregulation	Hepatocellular carcinoma [89], bladder cancer [90], small-cell lung carcinoma [91], GBM [92], retinoblastoma, and neuroblastoma [93]
*BIM*	DNA hypermethylation	Downregulation	Renal cell carcinoma and chronic myeloid leukemia [94]
	H3K27me3 repressive mark	Downregulation	Burkitt’s lymphoma [172]
*APAF-1*	DNA hypermethylation	Downregulation	Leukemia [95], melanoma [96], and gastric [97], bladder, and kidney cancer [98]
*XAF1*	DNA hypermethylation	Downregulation	Gastric and bladder cancer [99,100,101]
*BCL-2*	DNA hypermethylation	Downregulation	Prostate cancer [102]
	miR-15/16 silencing by histone deacetylation	Upregulation	Pituitary adenoma [180] and B-cell chronic lymphocyte leukemia [181]
	miR-34 hypermethylation	Upregulation	Gastric cancer, chronic lymphocytic leukemia, pancreatic, breast, colon, and kidney cancer, and Burkitt’s lymphoma [184,185]
*BAX*	DNA hypermethylation	Downregulation	Multiple myeloma cells [103] and Burkitt’s lymphoma [104]
	H3 and H4 deacetylation	Downregulation	Colon cancer [171]
*BAK*	DNA hypermethylation	Downregulation	Multiple myeloma cells [103] and Burkitt’s lymphoma [104]
*PUMA*	DNA hypermethylation	Downregulation	Multiple myeloma cells [103] and Burkitt’s lymphoma [104]
*BAD*	DNA hypermethylation	Downregulation	Multiple myeloma cells [103]
*BCL-2L10*	DNA hypermethylation	Downregulation	Gastric cancer [105] and leukemia [106]
*BIK*	DNA hypermethylation	Downregulation	Glioma [107], RCC [108], prostate cancer [109], and myeloma [110]
*BNIP3*	DNA hypermethylation	Downregulation	Gastric cancer [111], colorectal cancer [112], leukemia [113], and HCC [114]
*HRK*	DNA hypermethylation	Downregulation	Colorectal, gastric [115], GBM [116], PCNSL [117], and prostate cancer [118]

**Table 2:** Epigenetic modification of core apoptotic machinery by miRNAs.

miRNA	miRNA Status in Cancer	Target Gene & Outcome	Cancer Type
miR-29b	Downregulated	HRK upregulation	Lung [187], prostate [188], bladder [189], and ovarian cancers [190] and GBM [191]
miR-193a-3p, miR-512-5p, miR-153, miR-133B	Hypermethylated	MLC1 upregulation	AML [192], gastric tumors [193], GBM [194], and lung cancer [195]
miR-127	Hypermethylated	BCL-6 upregulation	Bladder, prostate, breast, and lung cancer and lymphoma [197]
miR-221, miR-222	Upregulated	PUMA and CASPASE-3 downregulation	Bladder [203] and glioma [204]
miR-17-92, miR-106b-93-25	Overexpressed	BIM downregulation	Lung, colon, lymphoma, medulloblastoma, and multiple myeloma [206,207]
miR-135a	Downregulated	BCL-xL upregulation	Hodgkin lymphoma, AML [210], and ovarian cancer [211]
miR-451	Downregulated	BCL-2 upregulation	Gastric cancer [216]

**Table 3:** Epigenetic modification of apoptosis regulatory pathways and genes.

Other Apoptosis Related Genes	Epigenetic Modification	Outcome
*DAPK*	DNA hypermethylation [121,122,123]	Downregulation
*HIC1*	DNA hypermethylation [124,125]	Downregulation
*p16^INK4a^*	DNA hypermethylation [119]	Downregulation
*APC*	DNA hypermethylation [119]	Downregulation
*TP53*	DNA hypermethylation [126]	Downregulation
*RASSF1*	DNA hypermethylation [127,128,129,130]	Downregulation
*MGMT*	DNA hypermethylation [119,120]	Downregulation
	Increasing H3K4me3 [35]	Downregulation
*BRCA1*	DNA Hypermethylation [119]	Downregulation
	Increased H3K4me3 [35]	Downregulation
*MLH1*	DNA hypermethylation [119]	Downregulation
	Decreased H3K4 methylation [158]	
*pS2*	DNA hypomethylation [147]	Upregulation
*HOX11*	DNA hypomethylation [148]	Upregulation
*c-MYC*	DNA hypomethylation [149,150]	Upregulation
*c-N-RAS*	DNA hypomethylation [149,150]	Upregulation
*MRE11*	H2A.X-Y142 phosphorylation [162,163]	Binding to γ-H2A.X sites is blocked
*RAD50*		
*NBS1*		
*53BP1*		
*BRCA1*		
*NF-κB*	H2B-S14ph [166]	Inhibited by reduced nuclear trafficking
*E2F1*	Protein acetylation [174]	Half-life and DNA binding affinity is increased
*KU70*	Protein acetylation [175]	Interaction with *BAX* is disrupted

**Table 4:** Epi-drugs under investigation.

Targeted Epigenetic Modification	Class	Agent	FDA Approval Status	Targeted Disease
**DNA** **Methylation**	DNMT1 inhibitors	Azacitidine	Approved (2004)	MDS and AML
		Decitabine	Approved (2006)	MDS and AML
		Guadecitabine	Clinical trial	Liver, pancreatic, bile duct, or gallbladder cancer (*NCT03257761*), non-small-cell lung cancer (*NCT03220477*), kidney cancer (*NCT03220477*), AML (*NCT02878785*), and urothelial cancer (*NCT03179943*)
		4-Thio-2-deoxycytidine (TdCyd)	Clinical trial	Solid tumors (*NCT02423057*)
	IDH1 and IDH2 inhibitors	AG-120 (Ivosidenib)	Clinical trial	AML (*NCT03173248*), myelodysplastic syndrome, chronic myelomonocytic leukemia (*NCT03564821*), and hematologic malignancies (*NCT03471260*)
		AG-221 (Enasidenib)	Clinical trial	AML (*NCT03013998, NCT02577406, NCT03825796, NCT03728335, NCT03683433, NCT03881735*) and MDS (*NCT03383575*)
		AG-881 (Vorasidenib)	Clinical trial	Hematologic malignancies (*NCT02492737*)
		IDH305	Clinical trial	Low-grade gliomas (*NCT02987010*)
**Histone** **Acetylation**	HDAC inhibitors	Vorinostat (SAHA)	Approved (2006)	Cutaneous or peripheral T-cell lymphoma
		Belinostat	Approved (2014)	Peripheral T-cell lymphoma
		Romidepsin	Approved (2009)	Cutaneous or peripheral T-cell lymphoma
		Trichostatin A	Clinical trial	Hematologic malignancies (*NCT03838926*)
		Panobinostat	Approved (2015)	Multiple myeloma
	Histone acetylation reader inhibitors	OTX015	Clinical trial	Hematologic malignancies (*NCT01713582*)
		ABBV-075 (Mivebresib)	Clinical trial	Breast cancer, non-small-cell lung cancer, AML, multiple myeloma, prostate cancer, small-cell lung cancer, and non-Hodgkin’s lymphoma (*NCT02391480*)
		BMS-986158	Clinical trial	Advanced cancers (*NCT02419417*), refractory solid tumors, central nervous system tumors, or lymphoma (*NCT03936465*)
		GSK2820151	Clinical trial	Advanced or recurrent solid tumors (*NCT02630251*)
**Histone** **Methylation**	HMT inhibitors	Tazemetostat	Clinical trial	Advanced solid tumors, non-Hodgkin’s lymphoma, histiocytic disorders (*NCT03213665*), synovial sarcoma (*NCT02601937*), urothelial carcinoma (*NCT03854474*), and ovarian or endometrial cancer (*NCT03348631*)
		CPI-1205	Clinical trial	Castration-resistant prostate cancer (*NCT03480646*)
		DS-3201 (Valemetostat)	Clinical trial	Lymphomas (*NCT02732275*), AML, and ALL (*NCT03110354*)
		GSK2816126	Clinical trial	Diffuse large B-cell lymphoma, transformed follicular lymphoma, other non-Hodgkin’s lymphomas, solid tumors, and multiple myeloma (*NCT02082977*)
		Pinometostat	Clinical trial	AML (*NCT03724084*, *NCT03701295*)
	HDM inhibitors	GSK2879552	Clinical trial	Small-cell lung carcinoma (*NCT02034123*) and AML (*NCT02177812*)
		INCB059872	Clinical trial	Ewing sarcoma (*NCT03514407*), solid tumors, and hematologic malignancy (*NCT02712905*)
**miRNA** **Regulation**	miRNA mimics	MesomiR-1	Clinical trial	NSCLC and MPM (*NCT02369198*)
		MRX34	Clinical trial	Hepatocellular carcinoma, renal cell carcinoma, and melanoma (*NCT01829971*)
	miRNA antagonist	MRG-106	Clinical trial	T-cell lymphoma (*NCT03713320*)

## Data Availability

Not applicable.
